# An Ontario Health (Cancer Care Ontario) Clinical Practice Guideline: Consolidation or Maintenance Systemic Therapy for Newly Diagnosed Stage II, III, or IV Epithelial Ovary, Fallopian Tube, or Primary Peritoneal Carcinoma

**DOI:** 10.3390/curroncol28020107

**Published:** 2021-03-01

**Authors:** Hal Hirte, Xiaomei Yao, Sarah E. Ferguson, Taymaa May, Laurie Elit

**Affiliations:** 1 Department of Oncology, McMaster University, Hamilton, ON L8S 4L8, Canada; hirteh@hhsc.ca; 2 Program in Evidence-Based Care, Ontario Health (Cancer Care Ontario), Toronto, ON M5G 2L7, Canada; 3 Department of Obstetrics and Gynecology, University of Toronto, Toronto, ON M5G 1A1, Canada; Sarah.Ferguson@uhn.ca (S.E.F.); Taymaa.May@uhn.ca (T.M.); 4 Division of Gynecologic Oncology, Princess Margaret Cancer Center, Toronto, ON M5G 2C1, Canada; 5 Department of Obstetrics and Gynecology, McMaster University, Hamilton, ON L8S 4L8, Canada

**Keywords:** clinical practice guideline, consolidation therapy, evidence-based, maintenance therapy, ovarian cancer, systemic therapy

## Abstract

Objective: To provide recommendations on systemic therapy options in consolidation or maintenance therapy for women with newly diagnosed stage II, III, or IV epithelial ovary, fallopian tube, or primary peritoneal carcinoma including all histological types. Methods: Consistent with the Program in Evidence-based Program’s standardized approach, MEDLINE, EMBASE, PubMed, Cochrane Library, and PROSPERO (the international prospective register of systematic reviews) databases, and four relevant conferences were systematically searched. The Working Group drafted recommendations and revised them based on the comments from internal and external reviewers. Results: We have one recommendation for consolidation therapy and eight recommendations for maintenance therapy. Overall, consolidation therapy with chemotherapy should not be recommended in the target population. For maintenance therapy, we recommended olaparib (Recommendation), niraparib (Weak Recommendation), veliparib (Weak Recommendation), and bevacizumab (Weak Recommendation) for certain patients with newly diagnosed stage III–IV epithelial ovarian, fallopian tube, or primary peritoneal carcinoma, respectively. We do not recommend some agents as maintenance therapy in four recommendations. We are unable to specify the patient population by histological types for different maintenance therapy recommendations. When new evidence that can impact the recommendations is available, the recommendations will be updated as soon as possible.

## 1. Introduction

Epithelial ovary, fallopian tube, and primary peritoneal carcinoma (EOC) are leading causes of death among gynecological cancers [1]. In 2020, 3100 women are estimated to be diagnosed with EOC, which will result in 1950 deaths in Canada [2]. However, around 70% of stage III and IV patients have a relapse within three years after completing the standard treatment strategies, which will lead to further deaths later [3]. Thus, whether consolidation therapy (defined as treatment being given after cancer has disappeared following the initial therapy) or maintenance therapy (defined as treatment being given to help prevent a cancer recurrence after it has disappeared following initial therapy, which may be given for a long duration) can increase survival and improve patients’ reported outcomes with acceptable adverse effects becomes an important clinical question [4].

The Working Group (including one medical oncologist: H.H.; three gynecologic oncologists: L.E., S.F., T.M.; and one methodologist: X.Y.) of the Ovarian Cancer Guideline Development Group in Ontario, in association with the Program in Evidence-Based Care (PEBC) of Ontario Health (Cancer Care Ontario) in Canada, decided to develop this clinical practice guideline to answer the following research questions.

## 2. Research Questions

Does consolidation or maintenance systemic therapy improve overall survival (OS), progression-free survival (PFS), and patient-reported outcomes (quality of life (QoL)), with acceptable adverse effects in the target population? If so, what is the optimal regimen for maintenance therapy (dose, schedule, and frequency)?

- In the target population, do patients with *BRCA1/2* mutation (somatic or germline mutation) or homologous-recombination deficiency (HRD) have different optimal regimens for maintenance therapy and outcomes compared with patients without *BRCA* 1/2 mutation or HRD?
- Do patients with different histological subtypes (low-grade serous, endometrioid, clear cell, mucinous, undifferentiated or unclassifiable) or different stages have different optimal regimens for maintenance systemic therapy and outcomes?

## 3. Target Population

This included patients with newly diagnosed stage II, III, or IV EOC after surgery and completion of adjuvant therapy (patients who needed neoadjuvant therapy before surgery qualified for this guideline as well).

## 4. Methods

The core methodology used to develop the evidentiary base was the systematic review. The PEBC is mandated to post its approved practice guidelines on the Cancer Care Ontario Web site (http://www.cancercare.on.ca/) for dissemination to Ontario oncologists [5].

### 4.1. Literature Search

The systematic review is published separately. Briefly, the MEDLINE, EMBASE, and the Cochrane Library (January 2003 to August 2019); PubMed (January 2018 to October 2019); main relevant guideline Web sites and annual conference meeting abstracts (January 2017 to October 2019) were searched. Preplanned study selection criteria were used to screen the literature retrieved.

### 4.2. Internal Review

For the guideline document to be approved, 75% of the content experts who comprise the Ovarian Cancer Guideline Development Group in Ontario must cast a vote indicating whether or not they approve the document, and of those that vote, 75% must approve the document. The voting process was performed by emails. Every member of the Ovarian Cancer Guideline Development Group was required to vote one of the three options for the recommendations: (1) I approve this document without further comments or with the following comments and changes; (2) I disapprove this document because the following reasons; (3) The guideline is not in my expertise.

The PEBC Report Approval Panel, a three-person panel with methodology expertise, must unanimously approve the document.

In addition, six patients, survivors, or caregivers participated in Patient and Caregiver-specific Consultation Group, reviewed copies of the draft recommendations, and provided feedback.

### 4.3. External Review

Feedback on the approved draft guideline is obtained from content experts and the target users through two processes. Through the Targeted Peer Review, several individuals with content expertise are identified by the Working Group and asked to review and provide feedback on the guideline document. Through Professional Consultation, relevant care providers and other potential users of the guideline are contacted and asked to provide feedback through a brief online survey.

## 5. Results

### 5.1. Literature Search Results

There were 12,675 citations from the medical databases search. A total of 27 trials from 40 full-text articles [3,6,7,8,9,10,11,12,13,14,15,16,17,18,19,20,21,22,23,24,25,26,27,28,29,30,31,32,33,34,35,36,37,38,39,40,41,42,43,44] and one additional trial from a conference abstract [16] were then analyzed.

### 5.2. Internal Review

The main comments from Patient and Caregiver-specific Consultation Group were: (1) Overall survival (OS) is a critical outcome and a strong recommendation should be made based on OS benefit for a therapeutic agent. (2) Whether patients’ quality of life would be impacted after taking or not taking a maintenance therapy is very important. Of the nine members of the Ovarian Cancer Guideline Development Group in Ontario, eight cast votes and one abstained, for a total of 89% response in January 2020. Of those that cast votes, eight approved the document (100%) but required revision based on their comments. The following key issues were raised by the Ovarian Cancer Guideline Development Group in Ontario and PEBC Report Approval Panel: (1) In the SOLO1 trial for olaparib and PRIMA trial for niraparib, to report the interim analysis result for OS would mislead the readers. (2) Could a stronger recommendation for olaparib be made based on the SOLO1 trial? (3) In the PRIMA trial for niraparib, there is an error, i.e., there was a non-HRD subgroup in the paper. (4) The ICON7 trial showed significant OS benefit in the pre-planned subgroup of high risk for use of bevacizumab. Can we make a strong recommendation? (5) This guideline focuses on consolidation and maintenance use rather than adjuvant and maintenance use. Why did you recommend bevacizumab and veliparib with adjuvant therapy, and then as a maintenance therapy, respectively?

### 5.3. External Review

After approval of the document at the internal review, the authors circulated the draft document to external review participants for review and feedback. Main concerns expressed in the written comments were below: (1) The guidelines do not discuss histological type and disease grade. (2) The guidelines over emphasize the risk of toxicity with Poly ADP ribose polymerase (PARP) inhibitors, particularly as discussed in the justification sections. (3) The term “first-line surgery” is odd. (4) These guidelines seem to undervalue the impact of very long PFS. (5) Can you get the same benefit by using a PARP inhibitor after first recurrence as maintenance?

The final guideline recommendations reflected the integration of feedback obtained through the external review processes with the document as drafted by the Working Group and approved by the Ovarian Cancer Guideline Development Group and the PEBC Report Approval Panel.

## 6. Recommendations, Key Evidence, and Interpretation of Evidence

We are unable to specify the patient population by histological types for different maintenance therapy recommendations. The majority of patients in the eligible studies are high-grade serous. When new evidence that can impact the recommendations is available, the recommendations should be updated as soon as possible. The definition of strength of recommendations for this guideline is listed in Table 1.

GRADE, Grading of Recommendations, Assessment, Development, and Evaluation.

## 7. Consolidation Therapy

### Recommendation 1 (Strength: Recommendation)

Consolidation therapy with chemotherapy is ***NOT*** recommended in the target population.

**Qualifying statement:** The investigated consolidation chemotherapy agents include epidoxorubicin alone, cisplatin alone, topotecan alone, paclitaxel alone, 5-fluorouracil plus cisplatin, and paclitaxel plus cisplatin and carboplatin.

**Key evidence:** The certainty of the aggregate study evidence for eight trials (nine full-text publications) was moderate to low based on the GRADE approach [45].

The SWOG-9701/GOG-178 trial reported that a monthly cycle of paclitaxel for 12 cycles, led to a longer PFS than that for three cycles, but there was no benefit in OS [24,25]. However, the trial did not have sufficient power to support its conclusion. Additionally, the After-6 Protocol 1 trial did not find a PFS or OS benefit from paclitaxel as consolidation therapy [31]. The four other trials did not identify any statistically significant results for PFS or OS for paclitaxel plus cisplatin and carboplatin, epidoxorubicin alone, 5-fluorouracil plus cisplatin, or cisplatin alone [10,28,34,40]. The SWOG-9701/GOG-178 trial [24,25] indicated greater Grade 3 or higher hematologic adverse effects in the experimental group. The other five studies did not report the adverse effect outcomes between two groups [10,28,31,34,40]. No trials reported QoL outcomes.

The AGO-OVAR 7 trial and MITO-1 trial examined topotecan consolidation therapy [13,33]. Both trials showed that compared with observation, topotecan consolidation therapy did not result in improved PFS or OS.

**Justification:** In this patient population, the evidence does not show survival benefit of consolidation therapy with additional chemotherapy after completion of adjuvant therapy. Rather, it can cause more adverse effects and is more costly. Therefore, the Working Group members recommend against using consolidation therapy with chemotherapy. The Patient Consultation Group agreed.

## 8. Maintenance Therapy

### 8.1. Agents Recommended

#### 8.1.1. Recommendation 2 (Strength: Recommendation)

Maintenance therapy with olaparib 300 mg twice a day by mouth for up to two years or until progression should be recommended in newly diagnosed stage III, or IV EOC patients with *BRCA1/2* mutation (somatic or germline), who are in complete remission or partial remission status after first-line therapy with cytoreductive surgery and adjuvant therapy.

**Qualifying statements:** Patients who have no evidence of disease at two years stopped using olaparib, but patients who have a partial response at two years can continue receiving it. The strength of recommendation will be reconsidered when OS data are available.

**Key evidence:** The certainty of evidence of two trials is high when evaluated using the GRADE approach. In the SOLO1trial [3], patients with *BRCA1*, *BRCA2*, or both mutations (somatic or germline), who took olaparib alone as a maintenance therapy had a higher PFS rate than those in the placebo group (60% vs. 27%, *p* < 0.01), and the sensitivity analysis of investigator-assessed PFS showed the difference was 36.1 months (49.9 vs. 13.8 months, *p* < 0.01) between the two groups. However, the final OS data are immature. Patients in the olaparib group had more anemia and any Grade 3 adverse effects. There was no clinically meaningful difference between the two groups for QoL at two years.

In the PAOLA-1 trial [36], patients received bevacizumab with platinum-based chemotherapy as adjuvant therapy, and all patients continued receiving bevacizumab for up to another 11 months or until progression. At the end of adjuvant therapy, patients with complete or partial remission were randomized to receive olaparib as maintenance therapy for 24 months versus placebo. Olaparib led to higher PFS compared with placebo. Data for OS are not yet available. Patients in the experimental group had more Grade 3 and more anemia adverse effects. No statistically significant difference was found for QoL. Subgroup analyses showed that patients with HRD had better PFS.

**Justification:** In the SOLO1 trial, although the final OS data are immature, the effect magnitude of olaparib for PFS is large (36-month difference between two groups) in patients with *BRCA1/2* mutation with manageable adverse effects. Thus, the Working Group makes “Recommendation” for olaparib at present instead of “Weak Recommendation” as suggested by the internal and external reviewers.

In the discussion section of the PAOLA-1 trial, the authors realized the potential contamination bias due to additional bevacizumab therapy and the lack of an arm with olaparib monotherapy. Thus, it is unclear whether olaparib maintenance therapy alone will have benefit in patients with HRD versus patients without HRD.

In the PAOLA-1 trial, we are unable to identify an additional desirable effect from bevacizumab; thus, we do not recommend olaparib plus bevacizumab as maintenance therapy at present.

#### 8.1.2. Recommendation 3 (Strength: Weak Recommendation)

Maintenance therapy with niraparib 200 to 300 mg by mouth daily for three years or until progression can be recommended in the target population.

**Qualifying statement:** The strength of recommendation will be reconsidered when OS data are available.

**Key evidence:** The certainty of evidence of the PRIMA/ENGOT-OV26/GOG-3012 trial is high [1]. The results indicated that niraparib led to higher PFS in all patients. The subgroup analyses showed that niraparib had PFS benefit among patients with HRD and patients without HRD, and patients with or without *BRCA1/2* mutation, compared with placebo. Thus, HRD or *BRCA1/2* mutation is not a confounder. However, the OS data are not yet mature. Compared with placebo, niraparib led to more Grade 3 or higher adverse effects on treatment-related adverse effects, anemia, neutropenia, and thrombocytopenia. There was no difference in QoL between the two groups.

**Justification:** Although niraparib significantly improved PFS in all patients, it increased the risk of adverse effects. Since the median follow-up duration in this trial is 13.8 months only and the OS data are immature, the Working Group members make a weak recommendation for use of niraparib at present. Less than 25% of the Ovarian Cancer Guideline Development Group and External Review members wanted to make “Recommendation” rather than “Weak Recommendation”. The Patients’ Consultation Group emphasizes the results of OS and agrees with “Weak Recommendation” at present.

#### 8.1.3. Recommendation 4 (Strength: Weak Recommendation)

Concurrent use of bevacizumab 7.5 mg/kg intravenously three-weekly with adjuvant therapy for six cycles and continued use for up to 12 cycles or until progression as maintenance therapy can be recommended in newly diagnosed high-risk stage III, or IV EOC patients.

**Qualifying statement:** The definition of high-risk stage III or stage IV patients in the eligible study (ICON7 trial) was defined as stage III with residual disease >1 cm, inoperable stage III, or stage IV EOC (total 30 [6%] inoperable stage III or IV patients).

**Key evidence:** The aggregate evidence certainty of two large randomized controlled trials (RCT) on bevacizumab was moderate.

In the ICON7 trial, patients received six cycles of adjuvant paclitaxel plus carboplatin and followed placebo as maintenance therapy, versus paclitaxel plus carboplatin plus concurrent bevacizumab followed by bevacizumab for 12 cycles or until disease progression, versus placebo [18,30,32,38]. No PFS or OS benefit was found in the bevacizumab group at median 4.1 years. Bevacizumab led to more Grade 3 or 4 adverse effects. The pre-planned subgroup analysis showed that among high-risk patients (defined as stage III with residual >1 cm or stage IV), bevacizumab led to longer PFS and OS. The *p*-value of 0.01 from the interaction test demonstrated the benefit of bevacizumab in the high-risk patients. Additionally, QoL measurements indicated a worse score in patients in the bevacizumab group. The subgroup analysis for histological subtypes found no benefit of bevacizumab for OS outcome in patients with low-grade serous tumors or clear cell tumors.

In the GOG-0218 trial [11,27,29,39], patients in the control group (CG) received paclitaxel and carboplatin for six cycles, plus placebo from cycle two to up to cycle 22; patients in the experimental group 1 (EG1) received paclitaxel and carboplatin from cycle two to cycle six, plus bevacizumab from cycle 2 to cycle 22; and patients in the experimental group 2 (EG2) received paclitaxel and carboplatin for six cycles, plus bevacizumab from cycle two to cycle six and then placebo from cycle 7 to up to cycle 22. Patients in EG1 had a better PFS result than those in CG, but the final results showed no benefit for OS at median 8.6 years. There was no benefit for either PFS or OS in the EG2 when compared with the CG. More GRADE 3 or 4 adverse effect in EG1. There were no significant differences across the three treatment groups for QoL. The subgroup analyses showed that patients with or without a *BRCA* mutation in the EG1 had greater PFS than those in the CG. Patients in the EG1 experienced greater PFS than those in the CG with stage III or IV, respectively; but bevacizumab only had OS benefit in patients with stage IV disease. Only the serous tumor subgroup rather than non-serous tumors had benefit for PFS, but not for OS for patients in EG1 compared with CG.

**Justification:** Both trials randomized patients before adjuvant chemotherapy. Since there was no statistical difference between EG2 and CG for PFS or OS, there is uncertainty about the utility of bevacizumab given concurrently with cytotoxic chemotherapy. These two RCTs used different doses for bevacizumab (7.5 mg/kg in the ICON7 trial and 15 mg/kg in the GOG-0218 trial). However, the lower dose would be favored because it could cause fewer undesirable effects and would cost less. Therefore, the Working Group members suggest using the lower dose of 7.5 mg/kg for bevacizumab. Less than 25% of Ovarian Cancer Guideline Development Group and External Review members wanted to make “Recommendation” rather than “Weak Recommendation”. After considering the above desirable and undesirable effects of the maintenance therapy, the certainty of evidence, health equity, acceptability, feasibility, generalizability in Ontario, and patient preference, the Working Group members make a weak recommendation.

#### 8.1.4. Recommendation 5 (Strength: Weak Recommendation)

Concurrent use of veliparib 150 mg twice a day by mouth with adjuvant therapy for six cycles, and continued use of 400 mg twice a day by mouth for 30 cycles as maintenance therapy can be recommended in newly diagnosed stage III, or IV EOC patients with HRD.

**Qualifying statement:** The strength of recommendation will be reconsidered when OS data are available.

**Key evidence:** The VELIA/GOG-3005 trial investigated the efficacy of veliparib given either concurrently with adjuvant chemotherapy for six cycles (EG2), or concurrently and as maintenance therapy after adjuvant chemotherapy for up to 36 cycles (EG1) and compared with adjuvant chemotherapy alone (CG) [12]. The certainty of evidence of the trial is moderate. At median 28-month follow-up, patients in EG1 had a higher PFS than patients in CG. There was no PFS benefit in EG2 when compared with CG. Veliparib led to more Grade 3 or 4 adverse effects including neutropenia, thrombocytopenia, nausea, and vomiting. No clinically significant difference was found for QoL. The subgroup analysis showed the PFS benefit in patients with *BRCA1/2* mutation when compared with patients without *BRCA1/2* mutation; intervention in EG1 led to higher PFS in patients with HRD and patients with stage III, rather than in patients with non-HRD or stage IV when comparing with intervention in CG, but the interaction test’s *p*-value was not statistically significant for both subgroup analyses.

**Justification:** Although veliparib showed benefits for PFS, no OS results are available at present and it has adverse effects. This trial randomized patients before adjuvant therapy and analyzed patients, including disease progression, after adjuvant therapy. It is not clear what the benefit of concurrent veliparib with adjuvant chemotherapy was. This EG2 did not demonstrate a PFS benefit compared with the CG. Additionally, since there was no maintenance-alone arm, it is unclear what benefit was conferred by EG1 as compared with veliparib given as a maintenance treatment alone. Only one trial is available for veliparib; therefore, the doses listed in the recommendation are derived from this RCT. Less than 25% of Ovarian Cancer Guideline Development Group and External Review members wanted to make “Recommendation” rather than “Weak Recommendation”. However, the Working Group members stay with a weak recommendation at present.

### 8.2. Agents Not Recommended

#### 8.2.1. Recommendation 6 (Strength: Recommendation)

Pazopanib should ***NOT*** be recommended for use as maintenance therapy in the target population.

**Key evidence:** The evidence certainty was moderate for the AGO-OVAR16 trial [14,17,21,41] and low for the East Asian Study [23]. In the AGO-OVAR16 trial, at median 24.3 months, pazopanib resulted in greater PFS, but no benefit for final OS analysis at seven years. Patients in the pazopanib group had more neutropenia, thrombocytopenia, and any Grade 3 or 4 adverse effects. QoL results were inconsistent. In the subgroup analyses, there was no desirable effect from pazopanib in patients with *BRCA1/2* mutation for PFS, but it led to benefits for patients without *BRCA1/2.* (17.7 vs. 14.1 months, *p* = 0.02)

Kim et al. combined patients from an East Asian Study with Asian patients from the AGO-OVAR16 trial [23]. No benefit was found for PFS, but a trend of worsening OS was found in the pazopanib group.

**Justification:** Although pazopanib can improve PFS in non-Asian patients without *BRCA1/2* (median improved time, 3.6 months), it has severe adverse effects, no benefit for OS and results in a worse outcome in Asian patients. The Patients’ Consultation Group was greatly concerned about the benefit versus harm. After considering the certainty of evidence, balance of the benefits and harms, and patient preference, the Working Group members recommend not to use pazopanib in the target population in Ontario.

#### 8.2.2. Recommendation 7 (Strength: Recommendation)

Maintenance therapy with interferon-alpha, erlotinib, abagovomab, oregovomab, or sorafenib, should ***NOT*** be recommended in the target population.

**Key evidence:** This group included seven trials with nine full-text publications [6,7,8,9,19,20,22,37,44]. The aggregate study evidence certainty was moderate to low.

The two trials did not find benefit of alpha-interferon for PFS or OS, respectively [6,20]. One trial did not indicate any benefit of erlotinib for PFS or OS. Worse QoL scores were reported in the erlotinib group [44]. The MIMOSA trial found no statistically significant difference for PFS, OS, or any serious adverse effects between the abagovomab and the placebo groups [37]. Another trial reported no statistically significant difference for time to relapse, OS, any serious adverse effects, and QoL between the oregovomab and the placebo groups [7,8,9]. Another two phase II trials showed that there was no benefit from sorafenib on PFS or OS, respectively [19,22].

**Justification:** From the existing evidence, the Working Group members believe that there are no benefits but some harms and more costs for the above maintenance therapy in newly diagnosed EOC patients. Thus, the Working Group recommends against using them. The Patients’ Consultation Group agrees with this recommendation.

#### 8.2.3. Recommendation 8 (Strength: Recommendation)

Concurrent use of nintedanib with adjuvant therapy and continued use as maintenance therapy should ***NOT*** be recommended in patients with newly diagnosed stage III with residual >1 cm or stage IV EOC.

**Key evidence:** The aggregate study evidence certainty in the AGO-OVAR12 trial was high [14,35]. At median five years, patients in the nintedanib group had a greater PFS, but the time difference was 1.0 month between the two groups. The subgroup analysis did not show statistical difference for PFS in high-risk patients, but nintedanib led to a higher PFS in non-high-risk patients. There is no benefit for OS. Patients in the nintedanib group had more Grade 3 or 4 adverse effects. In a conference abstract [16], the additional nintedanib led to worse PFS and worse OS results in patients with stage III or IV ovarian cancer, and increased any Grade 3 or 4 toxicity.

**Justification:** The Patients’ Consultation Group was concerned about the benefit against harm in this subgroup of non-high-risk patients. Additionally, the CHIVA trial showed worse survival results.

After considering the certainty of evidence, balancing the benefits and harms, and patient preference, the Working Group members recommend not using nintedanib.

#### 8.2.4. Recommendation 9 (Strength: Recommendation)

Concurrent use of lonafarnib, enzastaurin, or trebananib with adjuvant therapy and continued use as maintenance therapy should ***NOT*** be recommended in the target population.

**Key evidence:** One trial did not find any benefit of lonafarnib for PFS and OS compared with observation [26]. Another trial did not find that additional enzastaurin as an adjuvant and maintenance therapy could improve PFS when compared with no maintenance therapy [43]. The TRINOVA-3/ENGOT-OV2/GOG-3001 did not find benefit for PFS or OS outcomes [42]. No significant difference was reported for QoL. Their aggregate study evidence certainty was moderate to low.

**Justification:** From the existing evidence, the Working Group members found that there are no benefits, some harms, and more cost for the above maintenance therapy. Thus, the Working Group recommends not using these agents in the target population in Ontario.

## 9. Further Research

High-quality RCTs to investigate new maintenance agents, especially for those that can improve OS, are highly needed. These studies could also provide treatment guidance for different histological types or molecular subsets in the target population.

## 10. Guideline Limitations

The cost-effectiveness of therapy agents and test resource issues are beyond the scope of the PEBC guideline. The Working Group members leave resource consideration to other decision makers.

## Figures and Tables

**Table 1:** Strength of Recommendations for this Guideline (modified based on GRADE [45]).

Strength	Definition
Recommendation to use the intervention	The guideline Working Group * believes the benefits of the maintenance therapy in newly diagnosed stage II, III, or IV ovarian cancer patients clearly outweigh the harms for nearly all patients and the group is confident to support the recommended action.
Weak recommendation to use the intervention	The guideline Working Group * believes the benefits and harms of the maintenance therapy in the target population are closely balanced or are more uncertain but still adequate to support the recommended action.
No recommendation for the intervention	The guideline Working Group * is uncertain whether the benefits and harms of the maintenance therapy in the target population are balanced and does not recommend a specific action.
Weak recommendation against the intervention	The guideline Working Group * believes the benefits and harms of the maintenance therapy in the target population are closely balanced or are more uncertain but still adequate to support the recommended action.
Recommendation against the intervention	The guideline Working Group * believes the harms of the maintenance therapy in the target population clearly outweigh the benefits for nearly all patients and the group is confident to support the recommended action.
	The factors considered in the above judgments include desirable and undesirable effects of the maintenance therapy, the certainty of evidence, patient preference, health equity, acceptability, feasibility, and generalizability in Ontario.

* The guideline Working Group includes one medical oncologist, three gynecologic oncologists, and one guideline methodologist.

## Data Availability

Not applicable.
